# Comparison of Direct and Indirect *Toxoplasma gondii* Detection and Genotyping in Game: Relationship and Challenges

**DOI:** 10.3390/microorganisms9081663

**Published:** 2021-08-04

**Authors:** Kaya C. Stollberg, Gereon Schares, Anne Mayer-Scholl, Iryna Hrushetska, Susanne Diescher, Annette Johne, Martin H. Richter, Nadja S. Bier

**Affiliations:** 1 German Federal Institute for Risk Assessment (BfR), Department for Safety in the Food Chain, 10589 Berlin, Germany; kaya.stollberg@bfr.bund.de; 2 National Reference Laboratory for Toxoplasmosis, Institute of Epidemiology, Friedrich-Loeffler-Institut (FLI), Federal Research Institute for Animal Health, 17493 Greifswald, Insel Riems, Germany; gereon.schares@fli.de; 3 German Federal Institute for Risk Assessment (BfR), Department for Biological Safety, 12277 Berlin, Germany; anne.mayer-scholl@bfr.bund.de (A.M.-S.); iryna.hrushetska@bfr.bund.de (I.H.); susanne.diescher@bfr.bund.de (S.D.); annette.johne@bfr.bund.de (A.J.); martin.richter@bfr.bund.de (M.H.R.)

**Keywords:** toxoplasmosis, zoonosis, serology, pepsin digestion, magnetic capture, qPCR, bioassay, wild boar, deer, PCR-RFLP

## Abstract

The importance of game as a source of *Toxoplasma gondii* (*T. gondii*) infection in humans is largely unknown. New data on the presence of *T. gondii* in game hunted in the Federal State of Brandenburg, Germany, were obtained by direct and indirect detection (ELISA). DNA extracted either directly (5 g heart or foreleg muscle, DE) or after acid pepsin digestion (50 g heart, PD) or enriched by magnetic capture (50 g heart, MC) was examined by real-time PCR (qPCR). ELISA revealed seroprevalences of 20% in wild boar (*Sus scrofa*), 11% in roe deer (*Capreolus capreolus*) and 6% in red deer (*Cervus elaphus*). *T. gondii* DNA was detected by at least one direct detection method in 12% of wild boar, 6% of roe deer, 2% of fallow deer (*Dama dama*) and 2% of red deer. In both, positive wild boar and roe deer, *T. gondii* type II specific alleles were the most prevalent, as assessed by PCR-restriction fragment length polymorphism. The highest proportion of positive animals was detected by MC qPCR, followed by PD qPCR with a similar proportion of positive findings. Investigation of 50 g of heart muscle revealed a significantly higher proportion of positive qPCR results than analysis of 5 g (*p* = 0.048). An association between seropositivity and direct detection was evident in wild boar and roe deer (*p* < 0.001). Infectivity of *T. gondii* DNA–positive samples was confirmed by bioassay (4/4), providing evidence that game could represent a relevant source of viable *T. gondii* posing a risk for human infection.

## 1. Introduction

*Toxoplasma gondii* (*T. gondii*) is a zoonotic protozoan parasite with a broad host range that is thought to infect virtually all mammals and birds [1]. It is the cause of toxoplasmosis, one of the most common parasitic zoonoses worldwide [1]. The general assumption is that one-third of the worldwide human population is chronically infected with the parasite [2]. In Germany, antibodies against *T. gondii* have been found in 55% of the general population [3]. In Europe, clonal type I, II and III strains of *T. gondii* are predominantly present, with clonal type II strains and, to a lesser degree, clonal type III strains being the most common [4]. While acquired toxoplasmosis is mostly asymptomatic in immunocompetent persons, it can cause a more severe or even fatal course of disease in immunocompromised individuals, with chorioretinitis, encephalitis and pneumonia as potential outcomes [5,6]. Congenital toxoplasmosis can result in various symptoms in the unborn child, such as chorioretinitis, intracranial calcifications and hydrocephalus, and can also lead to miscarriage [7].

Human toxoplasmosis can occur by consumption of raw or undercooked meat of infected animals containing tissue cysts with *T. gondii* bradyzoites. The accidental ingestion of *T. gondii* oocysts through consumption of contaminated food or water or after contact to contaminated soil or cat feces is another possible route of infection. *T. gondii* tachyzoites are the causative agents of congenital infection, which can occur through transplacental transmission in the course of a primary infection during pregnancy. Transmission through organ transplantations or blood transfusions via tachyzoites or bradyzoites is also possible but rarely occurs [8,9]. 

There are considerable knowledge gaps regarding the relative importance of the different transmission routes of *T. gondii*. About 50% of *T. gondii* infections in the USA are assumed to be foodborne and *T. gondii* ranks among the most important foodborne pathogens in the USA and the Netherlands [10,11,12]. Additionally, consumption of raw or undercooked meat, including game, was linked to a higher risk of *T. gondii* infection in Europe [13,14]. Multiple known cases of acute toxoplasmosis have been associated with the consumption of raw or insufficiently heated deer and wild boar meat [15,16,17,18,19,20]. Handling raw meat or the carcasses of wild animals could also result in potential smear infections [21,22]. 

In Germany, hunting and, presumably, consumption of cloven-hoofed game, such as wild boar (*Sus scrofa*), roe deer (*Capreolus capreolus*), fallow deer (*Dama dama*) and red deer (*Cervus elaphus)*, has increased during the past twenty years [23]. Moreover, hunters as frequent consumers of game meat are assumed to have a higher risk for *T. gondii* infection and high seroprevalences have already been identified in hunters in Slovakia [23,24]. Furthermore, the presence of *T. gondii* has been found in a considerable proportion of venison from retail markets in Scotland intended for human consumption [25]. Nonetheless, there is no official meat hygiene regulation for a general examination of carcasses of livestock or game for *T. gondii* in Germany and data on the occurrence of *T. gondii* in game are scarce. 

Many studies focus on serological data, but the detection of antibodies only provides information on former exposure to the parasite. However, direct detection proves the actual presence of *T. gondii* in the investigated tissue and is thus needed to more reliably assess the risk associated with the consumption of game. Direct detection can be challenging due to the irregular distribution and often low concentration of tissue cysts in meat, e.g., estimated at one tissue cyst per 50–100 g of pork [26,27]. Therefore, the sensitivity of a method is dependent on the sample size and can be significantly affected when only small sized tissue samples are analysed. The mouse and especially cat bioassays are regarded as reference methods among direct detection methods for *T. gondii* [28]. They show a high sensitivity due to the large sample size analysed (50–100 g tissue artificially digested in mouse bioassay and up to 500 g directly fed to cats) and additionally offer information on infectivity [29]. However, from an ethical point of view, and due to the large amount of costs, time and work involved, the bioassay is rather ill-suited for the screening of large sample numbers. A great variety of sensitive molecular methods for the direct detection of *T. gondii* are available, such as real-time PCR (qPCR) or nested PCR (nPCR), but sensitivity might be limited if DNA is obtained by using commercially available kits that only allow the use of very small sample sizes, e.g., 25 mg. The combination of molecular detection of *T. gondii* DNA with parasitological methods, such as acid pepsin digestion, can be used to process larger sample sizes and to concentrate the bradyzoites released during the process. Furthermore, a sequence-specific magnetic capture (MC) allows specific concentration and isolation of *T. gondii* DNA with simultaneous elimination of inhibitory substances [28,29]. 

This is the first study gathering data on the occurrence of *T. gondii* by direct and indirect detection in wild boar, roe deer, fallow deer and red deer in Germany to evaluate the risk associated with the consumption of game. Moreover, concordance of three different direct detection procedures in combination with 529-bp repetitive element (RE) qPCR was determined and partially complemented by a mouse bioassay as reference method. Because data on genotypes are critical for the understanding of potential risks and zoonotic impact of *T. gondii*, we analysed PCR-positive tissue samples and viable *T. gondii* in vitro isolates by PCR-restriction fragment length polymorphism (PCR-RFLP) to assess prevailing clonal types in German game. This dataset was complemented with serological data from the present and a recently published study [30] to assess the correlation between seropositivity and the actual presence of *T. gondii* in meat. 

## 2. Materials and Methods

### 2.1. Samples and Sample Preparation 

Access to hunting areas was provided by the German Institute for Federal Real Estate (BImA). Wild boar (*S. scrofa*), roe deer (*C. capreolus*), fallow deer (*D. dama*) and red deer (*C. elaphus*) were sampled in four consecutive hunting seasons (October until January) of the years 2017–2020. All animals were made available for sampling post-mortem and were legally shot for human consumption during driven hunts organized by the German Federal Forest Service. No animal was killed for the purpose of sampling. For this study, a total of 635 animals (306 wild boar, 184 roe deer, 80 fallow deer and 65 red deer) from the German Federal State of Brandenburg were sampled. Fallow deer was only available in two hunting seasons of 2019/2020 and 2020/2021. Animals were categorized into three age groups, juveniles (<1 year old), yearlings (1–2 years old) and adults (>2 years old) as previously described by Bier et al. [30].

Blood was obtained and serum prepared as previously detailed [30]. Heart and foreleg muscle tissue were sampled, transported and stored at 4 °C before tendons, fat and connective tissue were removed using sterile forceps and single-use scalpels (in the following referred to as pure muscle tissue). Five grams of pure muscle tissue were taken in about 0.5–1 g cuts from different regions of the sample, chopped into ground pieces and stored at −20 °C until direct DNA extraction. For acid pepsin digestion (PD) and magnetic capture (MC), 50 g of pure heart muscle tissue were cut into 1 cm^3^ pieces and stored at 4 and −20 °C, respectively, until further analysis. The heart was sampled because it is known as one of the predilection sites for *T. gondii* in pigs, small ruminants, horses and poultry [31] and has been previously used in wildlife studies [32,33]. Foreleg muscle tissue was sampled as representative for skeletal muscle. As in most cases, only a small fraction of foreleg muscle was made available and sample preparation proved difficult and time-consuming due to the necessity to remove a high amount of tendons and connective tissue, it was only sampled during the hunting season of 2017/2018.

### 2.2. ELISA

The commercially available ELISA kit ID Screen^®^ Toxoplasmosis Indirect Multi-species (ID.Vet, Grabels, France) was used to detect *T. gondii*–specific antibodies as previously described [30] in sera from 126 wild boar, 59 roe deer, 80 fallow deer and 18 red deer collected in two hunting seasons between 2019 and 2021. Combined with already published results [30], the serological dataset includes results of all animals sampled in four consecutive hunting seasons between 2017 and 2021 (306 wild boar, 184 roe deer, 80 fallow deer and 65 red deer).

### 2.3. Acid Pepsin Digestion

Acid pepsin digestion was performed within seven days after sampling. Pure heart muscle tissue (Section 2.1) was ground to the consistency of minced meat, using a household chopper (La Moulinette DPA130, Tefal, Rumilly, France). A total of 50 g (±0.1 g) of the minced sample was weighed, transferred to a 500 mL flask and further processed as already described [31,34,35]. After digestion and washing the resulting pellet, 200 mg of the pepsin digest were transferred to a 2 mL reaction tube and stored at −20 °C until further DNA extraction. For analysis via bioassay, the pepsin digest was resuspended in 1 mL of DMEM containing penicillin (1.000 i.u.) and streptomycin (1.000 µg) [35]. 

### 2.4. Direct DNA Extraction from 5 g of Heart or Foreleg Muscle Tissue (DE)

Direct DNA extraction from 5 g of muscle tissue (DE) was performed by using a slightly modified protocol published by the European Reference Laboratory for Parasites (Instituto Superiore di Sanità (ISS), Rome, MI-12) [36]. After thawing, pure muscle samples (5 g, Section 2.1) were vortexed at maximum speed with 2.5 g (±0.3 g) of sterile glass beads (Ø 4 mm) and 10 mL of lysis buffer, including 40 mM Tris-HCl pH 8, 10 mM EDTA, 1% SDS and 0.4 mg/mL proteinase K (30 mAnson-U/mg; Carl Roth, Karlsruhe, Germany), with proteinase K added separately after vortexing. After incubation at 56 °C (±3 °C) for 16–18 h in a hybridization oven under rotation, DNA extraction of 200 µL of the resulting lysate was carried out by using the “tissue protocol” of the QIAamp DNA Minikit (QIAGEN, Hilden, Germany) starting with the addition 200 µL of AL buffer. A total of 100 μL of 56 °C warm molecular biology grade water was used in two consecutive steps for elution of DNA by incubation for 5 min, followed by centrifugation at 6.000× *g* for one minute. DNA was stored at 4 °C. In each analysis, 5 g of minced pork spiked with 10^6^ in vitro cultivated tachyzoites (*T. gondii* strain RH) or water were used as positive and negative extraction control, respectively. 

### 2.5. DNA Extraction from Pepsin Digest (PD)

DNA extraction from 200 mg pepsin digested heart muscle tissue (50 g, PD, Section 2.3) was performed using the “tissue protocol” of the QIAamp DNA Minikit (QIAGEN, Hilden, Germany) with adjusted volumes of 1.440 μL ATL buffer and 160 μL proteinase K solution. After incubation at 56 °C and brief centrifugation, 200 μL of the lysed sample was added to 200 μL AL buffer and further processed as described in Section 2.4. For each experiment, 200 mg pepsin digest of 50 g minced pork were spiked with either 10^5^ in vitro cultivated tachyzoites (*T. gondii* strain RH) or water and used as positive and negative extraction controls, respectively. 

### 2.6. Sequence-Specific Magnetic Capture (MC)

The magnetic capture procedure was performed by using a published protocol [29] with modifications, i.e., shortened incubation times, magnetic beads from another manufacturer and a modified DNA elution: 50 g of pure heart muscle tissue (Section 2.1) were thawed, homogenized and lysed with 125 mL of lysis buffer according to the original protocol. The lysate was centrifuged at 3.500× *g* for 20 min at 4 °C to more easily allow the separation of fat. Then 12 mL of the supernatant (lysate) was transferred to a 15 mL tube while the remaining lysate was stored at −20 °C for further genotyping. After inactivation of proteinase K and addition of streptavidin sepharose, the cooled sample was incubated at room temperature for 15 min rotating at 10 rpm. Following centrifugation at 3.500× *g* for 10 min at 4 °C, 10 mL of supernatant was transferred, mixed with 10 pmol of capture-oligonucleotides, heated to 95 °C for 15 min and incubated at room temperature for 15 min, while rotating at 10 rpm. Per sample, 20 µL of MagnaLink™ streptavidin magnetic beads (TriLink Biotechnologies, San Diego, CA, USA, 10 mg/mL) was washed three times in 1 mL of nucleic acid binding and wash buffer (B&W buffer; 50 mM Tris HCl pH 8.0, 150 mM NaCl, 0.05% Tween 20). After resuspension in a final volume of 100 µL B&W buffer, the washed beads were added to the sample and incubated at room temperature for 60 min while rotating at 10 rpm. Following horizontal incubation in a magnetic stand on an orbital shaker for 5 min, the supernatant was removed by using disposable Pasteur pipettes (Alpha Laboratories, Eastleigh, UK) and the beads were washed three times using B&W buffer. As our prior experience showed that DNA elution by heating the sample to 100 °C resulted in PCR inhibition, elution of DNA was carried out by increasing pH, i.e., by resuspending beads in 100 µL of 100 mM sodium hydroxide (1 min, room temperature). After incubation in a magnetic stand for 1 min, eluted DNA was transferred to a new tube and neutralised by adding 5.5 µL of hydrochloric acid (5% *v*/*v*). Following confirmation of pH neutrality by using pH indicator paper, DNA was stored at 4 °C. In each analysis, 50 g of pork spiked with either 10^5^ in vitro cultivated tachyzoites (*T. gondii* strain RH) or water were used as positive and negative controls.

Additionally, 50 g of pork and heart muscle tissue of red deer spiked with a tenfold dilution series of 10^7^–10^0^ and 10^6^–10^2^ of in vitro cultivated tachyzoites (*T. gondii* strain RH), respectively, were analysed to verify the limit of detection.

### 2.7. Real-Time PCR (qPCR) Targeting the 529-bp Repetitive Element (529-bp RE)

Two qPCRs with comparable performance targeting 529-bp RE, originally described in Talabani et al. [37] (DE qPCR and PD qPCR) and Opsteegh et al. [29] (MC qPCR), were conducted with modifications as described in Bier et al. [38]. Samples were tested in duplicates. To achieve highest sensitivity, 10 µL of DNA sample was used as template in both qPCRs. In case of inhibition, samples were retested at higher dilutions (1:2, 1:5, 1:10, 1:20 and 1:50, Figure 1). Based on pretests, the 1:5 dilution was chosen as the first dilution level for PD qPCR and no PD samples were tested undiluted or 1:2 diluted.

**Figure 1:**
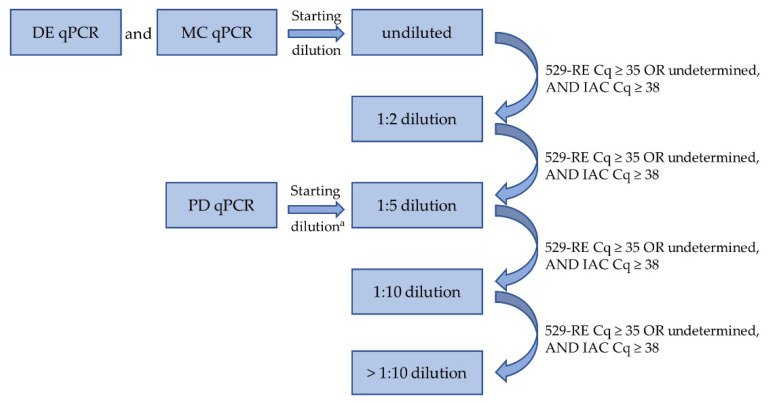
Methodology scheme for molecular detection of *Toxoplasma gondii*, showing different dilution levels used in qPCR. We used 10 µL of template DNA for the detection of *T. gondii* DNA via 529-bp repetitive element (RE) qPCR. DNA from direct DNA extraction (DE) from 5 g of muscle tissue was analysed via 529-RE qPCR (DE qPCR). DNA of 50 g of muscle tissue was examined by 529-RE qPCR after magnetic capture (MC qPCR). Another 50 g of muscle tissue was investigated via 529-RE qPCR after pepsin digestion (PD) and DNA extraction (PD qPCR). ^a^ Based on previous evaluation experiments, 1:5 dilution was selected as the first dilution level for PD qPCR.

All samples with exponential amplification and a Cq-value < 40 were scored positive for *T. gondii*, while all samples with a Cq-value ≥ 40 were scored negative, if amplification of the internal amplification control (IAC) revealed a Cq-value < 40. Samples with a Cq-value ≥ 38 and < 40 for the 529-bp RE were repeatedly tested to confirm the positive result. Samples with a Cq-value ≥ 35 or no amplification for the 529-bp RE in which the IAC showed a Cq-value ≥ 38 were considered inhibited and retested at the next higher dilution level (Figure 1).

### 2.8. Mouse Bioassay and In Vitro Cultivation

Fifty grams of pure heart muscle tissue (Section 2.1) from 23 animals were sent to the National Reference Laboratory for *T. gondii* in Germany, Friedrich-Loeffler Institut (FLI), for analysis via mouse bioassay after acid pepsin digestion (Section 2.3). Mouse bioassay was performed as already described [35]. Shortly, 2–4 IFNɣ-knockout mice (GKO, IFNɣ -/-, C.129S7(B6)-Ifngtm1Ts/J) per sample were inoculated subcutaneously using 500 µL of pepsin digested material. For strain isolation, the pleural cavity was flushed with 1 mL cell culture medium as well as homogenized brains and lungs of positively tested mice were inoculated on MARC-145 cells as previously described in Schares et al. [35]. 

### 2.9. Genotyping by Magnetic Capture and Conventional Endpoint PCR (cPCR) of the GRA6 Gene

For DNA samples that tested positive for 529-bp RE by MC qPCR, magnetic capture of the *GRA6* gene was additionally performed for genotyping. After thawing 12 mL of magnetic capture lysate, the capture of *GRA6* gene was performed as described above (Section 2.6), using 15 pmol of *GRA6* capture oligonucleotides (GRA6-CapF and GRA6-CapR) [29].

We used 10 µL of obtained DNA as template in a conventional endpoint PCR (cPCR), using a reaction mix with a total volume of 25 μL with 1× DreamTaq buffer (2 mM MgCl2), 0.2 mM of each deoxynucleoside triphosphate, 0.4 μM of each of the primers GRA6-F1 and GRA6-R1 [29] and 0.04 U/μL DreamTaq DNA polymerase (Thermo Fisher Scientific, Waltham, MA, USA). The cPCR reaction was performed on a thermal cycler (2720 Thermal Cycler; Applied Biosystems, Waltham, MA, USA) and initiated by a heat activation step of 4 min at 94 °C, followed by 45 amplification cycles (30 s at 94 °C, 30 s at 63 °C, and 45 s at 72 °C) and a final extension step of 10 min at 72 °C. PCR products (5 μL) were separated on 1.5% agarose by electrophoresis and visualized by using 0.08 µL/mL GelRed^®^ (Biotium, Fremont, CA, USA).

### 2.10. Genotyping by PCR-Restriction Fragment Length Polymorphism (PCR-RFLP)

*T. gondii* isolates obtained by mouse bioassay were genotyped by PCR-RFLP, as previously described [39], based on 12 genetic markers (SAG1, 5’-SAG2, 3’-SAG2, SAG3, BTUB, GRA6, c22-8, c29-2, L358, PK1, alt.SAG2 and Apico). 

DNA tissue samples that tested positive in DE qPCR and PD qPCR were analysed in a first experiment that used multiplex multilocus nested PCR-RFLP (Mn-PCR-RFLP) as described by Su et al. [40] with slight modifications (Supplementary Materials Table S1). Briefly, in the multiplex PCR reaction, 10 µL of DNA was used as template in the dilution that yielded the lowest Cq-value and least PCR inhibition in the 529-bp RE qPCR to increase sensitivity. DreamTaq DNA polymerase (Thermo Fisher Scientific, Waltham, MA, USA) was used in the nested PCR. In a second set of experiments, a modified protocol was performed to increase sensitivity and specificity. Briefly, a singleplex PCR was performed for each marker using undiluted, 1:2 and 1:5 diluted template DNA and PCR-cycling conditions were modified (Supplementary Materials Table S1). In the case of ambiguous *GRA6* PCR-RFLP profiles, the nested PCR product was sequenced.

In each PCR reaction, 100 genome equivalents of reference strains of the three clonal types I, II and III (strains RH, ME49 and NED) were included as positive controls. PCR products were digested by using one unit of restriction endonucleases (New England Biolabs, Ipswich, MA, USA) in accordance with Su et al. [40], with the exception of c22-8, for which the isoschizomere BcoDI was used. If incomplete digestion was observed, the amount of restriction enzyme was increased (up to two units of restriction enzyme per reaction) and incubation time was doubled. Digested PCR products (5 μL) were separated on 3% agarose gels in the presence of 0.08 µL/mL GelRed^®^ (Biotium, Fremont, CA, USA).

### 2.11. Data Analysis

Cohen’s kappa (κ) was used to determine the agreement between different methods. The strength of concordance was considered as follows: ≤0 = poor, 0.01–0.2 = slight, 0.21–0.4 = fair, 0.41–0.6 = moderate, 0.61–0.8 = substantial and 0.81–1 = (almost) perfect [41]. Fisher’s exact test was performed to assess a possible association between the direct detection of *T. gondii* DNA and *T. gondii*–specific antibodies in the sampled animals, as well as to other parameters such as sex, age, hunting season and sample size. Statistical analyses considering age were performed using three (<1 year old, 1–2 years old and >2 years old) age groups. A value of *p* < 0.05 was considered statistically significant. To assess the strength of the association, the odds ratios (ORs) were calculated. Calculations were performed using SPSS version 26 (SPSS Inc., Chicago, IL, USA) and an online tool (https://www.graphpad.com/quickcalcs/ by GraphPad Software, San Diego, CA, USA, accessed on 11 May 2021).

## 3. Results

### 3.1. Indirect Detection

During the hunting seasons 2019/2020 and 2020/2021, *T. gondii*–specific antibodies were detected in 14.3% (18/126; 95% CI: 8.7–21.6%) of wild boar, 6.8% (4/59; 95% CI: 1.9–16.5%) of roe deer and 5.6% (1/18; 95% CI: 0.1–27.3%) of red deer. No *T. gondii*–specific antibodies were found in fallow deer (0/80; 95% CI: 0–4.5%). Two samples from wild boar showed repeatedly doubtful results and were subsequently treated as negative. 

Combined with already published results, overall seroprevalences of 20.3% (62/306; 95% CI: 15.9–25.2%) in wild boar, 10.9% (20/184; 95% CI: 6.8–16.3%) in roe deer and 6.2% (4/65; 95% CI: 1.7–15%, Supplementary Materials Table S2) in red deer were determined in the four consecutive hunting seasons between 2017 and 2021. 

Considering the whole serological dataset, seropositivity increased with age for all investigated species. However, this increase was only statistically significant for wild boar and roe deer (*p* = 0.004 and < 0.001, respectively). No correlation between sex and seropositivity was found (*p* = 0.09–1, Supplementary Materials Table S2). Variations between seroprevalences of different hunting seasons were negligible (*p* = 0.12–1, Supplementary Materials Table S2).

### 3.2. Direct Detection of T. gondii in Muscle Tissue and Correlation with Serological Status

#### 3.2.1. Molecular Analysis of 5 g Muscle Tissue by DE qPCR

Heart muscle: The examination of all available heart muscle tissue samples by DE qPCR revealed *T. gondii* DNA–positive reactions in 7.6% (18/237; 95% CI: 4.6–11.7) of wild boar and 4.8% (7/146; 95% CI: 2–9.6) of roe deer samples, while fallow and red deer samples did not yield positive results (0/51; 95% CI: 0–7 and 0/52; 95% CI: 0–6.9, respectively; Table 1). Cq-values for *T. gondii* of these 25 positive samples ranged from 30.7 to 39.7. PCR inhibition was apparent in 83% (405/486) of the initial analyses using 10 µL undiluted sample DNA. However, four of all 25 positively tested samples had high Cq-values (≥34) and thus would probably not have been detected by using lower template concentrations. Valid results not affected by PCR inhibition, could be achieved by using a 1:2 dilution in 32% (155/486) and a 1:5 dilution in 29% (139/486) of samples, while the remaining 23% had to be retested in a 1:10 (*n* = 99), 1:20 (*n* = 6) or 1:50 dilution (*n* = 6).

Foreleg muscle: When DNA extracted from foreleg muscle tissue was examined, *T. gondii* was detected in one wild boar (7.7%; 1/13; 95% CI: 0.2–36) and in none of the roe and red deer samples (0/5; 95% CI: 0–52.2 and 0/7; 95% CI: 0–41). Of 22 animals that were analysed in both sample matrices, one wild boar and one roe deer were positive in heart muscle tissue only, while one wild boar sample was positive in both matrices. In wild boar and for all examined game species in total, the agreement between qPCR results of heart and foreleg muscle tissue was considered substantial and moderate (κ = 0.62 and 0.46, respectively; Supplementary Materials Table S3).

Association between seropositivity and DE qPCR: Analysis of the correlation between seropositivity and the presence of *T. gondii* in heart muscle tissue revealed that detection of *T. gondii* DNA by DE qPCR in wild boar and roe deer was significantly higher in seropositive animals (*p* < 0.001, Table 2). In more detail, 37% (17/46; 95% CI: 23.2–52.5) of seropositive wild boar and 41.2% (7/17; 95% CI: 18.4–67.1) of seropositive roe deer tested positive, while no *T. gondii* DNA was detected in seropositive red deer (0/4; 95% CI: 0–60.2). All seronegative animals also yielded negative results in DE qPCR, with the exception of one seronegative wild boar that tested positive for *T. gondii* DNA (0.5%; 1/191; 95% CI: 0.0–2.9, Table 2 and Supplementary Materials Table S6). 

A moderate agreement between DE qPCR and ELISA results in wild boar and roe deer (κ = 0.47 and 0.55, respectively; Table 3) was observed.

#### 3.2.2. Molecular Analysis of 50 g Heart Muscle Tissue by PD qPCR

Detection of *T. gondii* DNA in samples obtained by concentration of parasitic stages using pepsin digestion of 50 g heart muscle tissue (PD qPCR) revealed a proportion of positive animals of 11.7% (15/128; 95% CI: 6.7–18.6) in wild boar and 3.8% (3/79; 95% CI: 0.8–10.7) in roe deer. In contrast, no fallow and red deer samples tested positive (0/42; 95% CI: 0–8.4 and 0/31; 95% CI: 0–11.2, respectively; Table 1). Real-time PCR analysis revealed *T. gondii* Cq-values between 28.7 and 37.2. Valid results were achieved by using sample DNA at a 1:5 dilution (i.e., the starting dilution in case of PD qPCR) as template in 78% (219/280) of samples, while the remaining 22% (61/280) had to be retested in a 1:10 (*n* = 60) or a 1:50 dilution (*n* = 1) to avoid PCR inhibition.

Association between seropositivity and PD qPCR: In wild boar and roe deer, *T. gondii* DNA was detected in a significantly higher proportion in seropositive animals, than in seronegative individuals (*p* < 0.001, Table 2). While 61.9% (13/21; 95% CI: 38.44–81.89) of seropositive wild boar and 37.5% (3/8; 95% CI: 8.5–75.5) of seropositive roe deer also tested positive for *T. gondii* DNA in PD qPCR, no *T. gondii* DNA was detected in seropositive red deer (0/3; 95% CI: 0–70.8). Only two seronegative wild boar (1.9%; 2/107; 95% CI: 0.2–6.6) yielded positive PD qPCR results. The concordance of PD qPCR and ELISA results was substantial in wild boar (κ = 0.68) and moderate in roe deer (κ = 0.52, Table 3).

#### 3.2.3. Molecular Analysis of 50 g Heart Muscle Tissue by MC qPCR

By sequence-specific capture and detection of *T. gondii* DNA from 50 g heart muscle tissue (MC qPCR) the presence of *T. gondii* DNA was demonstrated in 13.2% (5/38; 95% CI: 4.4–28.1) of wild boar, 25% (2/8; 95% CI: 3.2–65.1) of roe deer, 3.5% (1/29; 95% CI: 0.1–17.8) of fallow and 8.3% (1/12; 95% CI: 0.2–38.5, Table 1) of red deer samples. Cq-values for *T. gondii* ranged between 30.8 and 38.5. 

An analysis of 50 g of pork spiked with in vitro cultivated tachyzoites revealed a limit of detection for MC qPCR of 10^2^ tachyzoites/50 g in 2/3 technical qPCR replicates and 10^3^ tachyzoites/50 g in 3/3 technical qPCR replicates. A lower sensitivity of 10^4^ in 3/3 technical qPCR replicates was obtained in the analysis of spiked heart muscle tissue from red deer.

Association between seropositivity and MC qPCR: The proportion of qPCR-positive samples was higher in seropositive animals than in seronegative animals for all species. However, this difference was only statistically significant in wild boar (*p* = 0.02, Table 2). In 50% of seropositive wild boar (3/6; 95% CI: 11.8–88.2) and roe deer (1/2; 95% CI: 1.3–98.7), as well as in 100% of seropositive red deer (1/1; 95% CI: 2.5–100), *T. gondii* DNA was detected. Two seronegative wild boar (6.3%; 2/32; 95% CI: 0.8–20.9), one seronegative roe deer and one seronegative fallow deer (16.7%; 1/6; 95% CI: 0.4–64.1 and 3.5%; 1/29; 95% CI: 0.1–17.8, respectively) yielded positive MC qPCR results. In wild boar and roe deer, ELISA and MC qPCR results demonstrated moderate (κ = 0.47) and fair (κ = 0.33) concordance, respectively (Table 3). ELISA and MC qPCR results in red deer showed perfect concordance (κ = 1, Table 3).

#### 3.2.4. Bioassay

Mouse bioassay revealed that hearts from three out of ten wild boar (30%; 3/10; 95% CI: 6.7–65.3) and from one out of thirteen roe deer (7.7%; 1/13; 95% CI: 0.2–36, Table 1) harboured infective *T. gondii*. No fallow or red deer samples were available for analysis via bioassay. From all four bioassay-positive animals, *T. gondii* strains could be isolated and were available for in vitro cultivation and further genotyping by PCR-RFLP (isolates 19–171, 20–528, 20–531 and 19–186).

As samples were partly preselected for bioassay analysis according to their serostatus, it was not justified to assess concordance of ELISA and bioassay results. In summary, three out of five seropositive wild boar and one out of three seropositive roe deer yielded a positive bioassay result. No seronegative animals (0/15) tested positive in bioassay.

#### 3.2.5. Overall Detection with at Least One Direct Detection Method 

Overall, *T. gondii* DNA was detected in heart muscle samples of 11.8% (28/237; 95% CI: 8–16.6) of wild boar, 5.5% (8/146; 95% CI: 2.4–10.5) of roe deer, 2% (1/51; 95% CI: 0.1–10.5) of fallow deer and 1.9% (1/52; 95% CI: 0.1–10.3) of red deer in at least one direct detection method (i.e., by qPCRs using various sample types, sample sizes and procedures, including also bioassay) (Table 1). In accordance with serological results, detection of *T. gondii* DNA in heart muscle significantly increased with age in wild boar and roe deer (*p* = 0.001 and 0.02, respectively; Supplementary Materials Table S4), while no correlations between sex or hunting season were observed (*p* = 0.23–0.48 and *p* = 0.08–0.58, respectively; Supplementary Materials Table S4). 

Association between seropositivity and qPCR results: Detection of *T. gondii* with at least one direct detection method was significantly higher in seropositive than in seronegative individuals for wild boar and roe deer (*p* < 0.001, respectively; Table 2). Among seropositive animals, 52.2% (24/46; 95% CI: 37–67.1) of wild boar, 41.2% (7/17; 95% CI: 18.4–67.1) of roe deer and 25% (1/4; 95% CI: 0.6–80.6) of red deer yielded positive results in at least one direct detection method. In seronegative animals, the presence of *T. gondii* was observed in four wild boars (2.1%; 4/191; 95% CI: 0.6–5.3), one roe and one fallow deer (0.8%; 1/129; 95% CI: 0.0–4.2 and 2%; 1/51; 95% CI: 0.1–10.5) by at least one direct detection method. Concordance between ELISA results and the summarized findings of all three direct detection methods was moderate in wild boar and roe deer (κ = 0.59 and 0.53, respectively) and fair in red deer (κ = 0.38, Table 3).

#### 3.2.6. Comparison between Different Direct Detection Methods 

A direct comparison between the individual methods was only possible for animals that had been tested by all three direct detection methods (DE, PD and MC qPCR; *n* = 87). In this comparison, MC qPCR detected *T. gondii* DNA in the highest proportion of animals (10.3%), followed by PD qPCR (8%) and DE qPCR (3.4%). Of the eleven positively tested animals (Supplementary Materials Table S5), only three were positive in all three direct detection methods, and two yielded positive results in PD qPCR and MC qPCR, whereas four and two animals tested positive exclusively in MC qPCR or PD qPCR, respectively (Supplementary Materials Tables S5 and S6). In total, moderate agreement between the individual methods (κ = 0.47–0.59) was observed, with PD qPCR and MC qPCR showing the highest (κ = 0.59) and DE qPCR and MC qPCR the least concordance (κ = 0.47, Table 4). While the investigation of 50 g of heart muscle tissue yielded a significantly higher proportion of positive qPCR results than the analysis of 5 g heart muscle tissue (*p* = 0.048), differences in positivity between individual methods were not statistically significant (*p* = 0.132–0.794, Table 5).

The proportion of positive results of the three methods and their agreement differed depending on animal species. In more detail, the highest proportion of positive tests for *T. gondii* DNA in wild boar (*n* = 38) was observed when samples were analysed by PD qPCR (15.8%), while lower proportions were observed by MC qPCR (13.5%) and DE qPCR (5.3%). Subsequently, MC qPCR and PD qPCR showed substantial agreement (κ = 0.68), while concordance between DE qPCR and PD qPCR or MC qPCR was moderate (κ = 0.46 and 0.54, respectively; Table 4). 

In roe deer (*n* = 8), MC qPCR revealed a higher percentage of *T. gondii* DNA–positive animals (25%) than the DE qPCR and PD qPCR, which showed identical results (12.5%, Table 6). Consequently, agreement between DE qPCR and PD qPCR was perfect (κ = 1), whereas the agreement between MC qPCR and DE qPCR or PD qPCR was moderate (κ = 0.6, respectively; Table 4). Among fallow deer (*n* = 29) and red deer (*n* = 12), only one individual each tested positive by MC qPCR (3.5% and 8.3%, respectively; Table 6), while none of the animals yielded positive results in the DE qPCR and PD qPCR (Table 6). 

An additional analysis of 23 animals, using the mouse bioassay as a reference, revealed that 100% of animals that yielded positive results in DE qPCR (4/4; Cq 31.9–34.8) and PD qPCR (3/3; Cq 30.1–31.9) also tested positive in the bioassay. The only sample available for examination by bioassay and MC yielded negative results in both methods. 

Association between seropositivity and qPCR results of different direct detection methods: Detection of *T. gondii* in seropositive and seronegative animals was consistent with previously described results (Section 3.2.1, Section 3.2.2 and Section 3.2.3, Table 2 and Table 6). In wild boar, DE qPCR and MC qPCR displayed the least, and PD qPCR the best agreement with ELISA results (κ = 0.46, 0.47 and 0.6; Supplementary Materials Table S7). Agreement between PD qPCR, or DE qPCR, and ELISA results in roe deer was moderate (κ = 0.6, respectively; Supplementary Materials Table S7). One seropositive wild boar and one red deer sample were exclusively tested positive, using PD qPCR and MC qPCR, respectively (Supplementary Materials Table S5). In seronegative animals, PD qPCR detected the lowest (0–6.3%) and MC qPCR the highest proportion of animals positive in *T. gondii* qPCR (0–16.7%), irrespective of the game species (Table 6). No seronegative animal tested positive by DE qPCR.

### 3.3. Genotypes

#### 3.3.1. Genotyping by Magnetic Capture and cPCR of the *GRA6* Gene

For nine samples that tested positive in MC qPCR (Cq-values: 30.8 to 38.5), magnetic capture of the *GRA6* gene was performed. However, none of the samples could be amplified in cPCR. An analysis of 50 g of pork spiked with 10^4^ to 10^7^ in vitro cultivated tachyzoites (*T. gondii* strain RH) revealed a limit of detection for MC *GRA6*-cPCR of 10^6^ tachyzoites/50 g. 

#### 3.3.2. Genotyping of *T. gondii* by PCR-RFLP

Three *T. gondii* isolates from wild boar and one isolate from roe deer could be typed in all twelve genetic markers as clonal type II Apico variant I (ToxoDB #3).

In addition, Mn-PCR-RFLP was performed directly on DNA of 34 tissue samples that tested positive for *T. gondii* DNA in DE qPCR and/or PD qPCR. Due to low sensitivity and occurrence of unspecific products, especially in alt.SAG2 and L358, a second set of experiments, using singleplex PCR, was performed, resulting in one sample that could be successfully genotyped in all twelve markers as clonal type II with type I allele at the Apico locus (ToxoDB #3, 19–201, Table 7). However, the majority of samples (79.4%; 27/34) could only be typed in less than nine genetic markers and two samples were not typeable in any marker. Typing of L358 (26/34) and 3´-SAG2 (25/34) was the most successful, while c29-2 (6/34) was rarely amplified.

## 4. Discussion

Cases of acute toxoplasmosis and two recent outbreaks among hunters in Wisconsin and Illinois, USA, have already been linked to the consumption of insufficiently heated or raw game [15,16,17,18,19,20,42]. In order to reliably assess the risk of human *T. gondii* infection from the consumption of game, information on the occurrence of *T. gondii* in the most frequently consumed game species is necessary. 

Generally, observed prevalences of *T. gondii* in wildlife vary widely across Europe. This heterogeneity can have multiple causes, such as the usage of different methods and parameters such as cutoffs, sample types and sampling strategy [43,44,45]. The geographic location with the local felid population density and climate prevailing in the region may also influence the detected prevalences [46,47,48].

The overall seroprevalence of 20.3% in wild boar, combined from this and our previous study [30], falls midrange of previously reported data for Germany (15–33%) [49,50,51] and Europe (6.7–56.6%) [52,53]. In roe deer, the determined seroprevalence of 10.9% was found to be lower than the 29% seroprevalence from another study in Germany [54] and was also in the lower range of seroprevalences described for Europe (2–60%) [55,56]. The proportion of *T. gondii*–specific antibodies in this study for red deer (6.2%) was low but ranged within seroprevalences reported for Europe (7.7–45%) [57,58]. To the best of our knowledge, this was the first investigation of fallow deer for the presence of *T. gondii*–specific antibodies in Germany. While no antibodies were found in this study, generally lower seroprevalences of 1% to 17% are reported in other European studies [56,58].

Most studies on the presence of *T. gondii* in game focus on seroprevalence, while direct detection of the parasite is only rarely performed. While serological data offer information on the presence of *T. gondii*–specific antibodies in game and thus on a former contact to the parasite, direct detection, on the other hand, proves the actual presence of parasitic stages in examined tissues. 

In our study, 11.8% of wild boar heart muscle samples tested positive for *T. gondii* DNA in at least one of the direct detection methods, which was comparable to a previous study in Italy wherein 16.2% tested positive in skeletal muscle [59]. In line with studies from Italy (2.4%) [59] and Ireland (4.2%) [60], only a few samples of deer (2–6%) tested positive for *T. gondii* DNA. In contrast, a study in Spain reported higher prevalences of 47.6% in fallow deer and 18.2% in red deer by investigation of 10 g of heart muscle tissue and brain using pepsin digestion and nested PCR [61], based on *B1* gene, which is reported to exist 35 times in the *T. gondii* genome [62,63]. As in the present study, larger sample sizes (50 g) were examined with pepsin digestion and magnetic capture in combination with highly sensitive 529-bp RE qPCRs, targeting a sequence estimated to exist in 200–300 copies per *T. gondii* genome [64], the lower prevalence might be caused by spatial differences, rather than a lack of sensitivity of the used methods. Moreover, the magnetic capture procedure used in this study showed a sensitivity of 10^2^ tachyzoites per 50 g of pork, comparable to the originally described protocol [29]. Nonetheless, higher sample numbers for fallow and red deer should be investigated to increase representativeness.

Molecular detection proves the existence of *T. gondii* parasitic stages in examined tissues, but offers no information on their infectivity. However, all of the samples that were positive for *T. gondii* DNA and that were also investigated by bioassay were able to successfully infect mice. Despite the low sample number investigated by bioassay, this suggests that the qPCR-positive samples observed in this study might at least partly contain infectious *T. gondii*. 

PCR-RFLP for genotyping of positively tested tissue samples proved difficult, probably due to low concentrations of *T. gondii* DNA, indicated by high Cq-values for *T. gondii* in the samples. Even though genotyping was only partially successful, type II alleles were the most often identified alleles among all typeable markers. Thus, type II seems to be the prevailing clonal type in cloven-hoofed game in the studied area, which is also predominant in the sylvatic environment in Europe [4]. However, type I and III alleles were also identified in individual markers implying the presence of mixed or atypical clonal types in game that could potentially be more virulent [65,66,67,68]. Atypical genotypes have already been identified in cats and non-canonical allele patterns were found in rodents and foxes in Germany, as well as in venison in Scotland [25,39,69]. As only few samples and isolates could be fully genotyped, further investigations are needed to assess prevailing clonal types in wild boar and deer in Germany.

Irrespective of indirect or direct detection, prevalences of *T. gondii* were generally lower in deer than in wild boar. This might be partly caused by a higher resistance of deer to *T. gondii*, as seen in other ruminants, such as cattle [70], or due to differences in diet. As herbivores, deer are mainly infected through the consumption of contaminated food and water and thus could be considered as good indicators of *T. gondii* oocysts in the environment [59,71]. In contrast, as opportunistic omnivores, wild boar have potentially more frequent contact with *T. gondii*.

A significant correlation between seropositivity and the presence of *T. gondii* parasitic stages could be determined in wild boar and roe deer, as the proportion of animals positive for *T. gondii* DNA was significantly higher in seropositive than in seronegative animals. Thus, seropositivity might be a good indicator for the presence of *T. gondii* tissue cysts in these game species. In fallow and red deer, the correlation of seropositivity and the actual presence of *T. gondii* in tissue samples could not be reliably assessed due to low sample numbers. In wild boar, roe deer and fallow deer, few seronegative samples also repeatedly tested positive for *T. gondii* DNA with one wild boar yielding positive results even in both, PD qPCR and MC qPCR. Therefore, the presence of tissue cysts in seronegative animals cannot be ruled out. Notably, methods that examined 50 g of muscle tissue identified more seronegative samples as positive for *T. gondii* DNA, which might be due to a higher sensitivity of these methods. However, as the true infection status of the sampled animals is unknown and no bioassay analyses were available for verification, it remains unclear if these are true-positive qPCR results.

Regarding the direct detection of *T. gondii*, one of the most important factors influencing sensitivity is the examined sample size, as *T. gondii* tissue cysts are sparse and irregularly distributed [8,32]. Thus, examination of larger sample sizes increases the probability of detection. In fact, analysis of 50 g of heart muscle tissue by PD and MC qPCR detected significantly higher proportions of *T. gondii* DNA than the DE qPCR of 5 g heart muscle tissue. In the literature, MC qPCR is generally accepted as one of the most sensitive molecular methods and has thus been used to analyse predilection sites of *T. gondii* [72,73,74]. In this study, MC qPCR was indeed able to detect the highest proportion of animals positive for *T. gondii* DNA. Unfortunately, as discussed above, no samples examined by MC qPCR where available for bioassay analysis to verify these results as true-positives. PD qPCR revealed comparable results to MC qPCR as was already reported in studies on chicken [75] and differences were occasionally observed in individual animals.

Molecular analysis of DNA samples obtained from heart muscle tissue, in combination with high template DNA concentration, frequently led to PCR inhibition in DE and PD qPCR. Among other reasons, this might be caused by high concentrations of hemoglobin or other PCR inhibitors or simply by the high concentration of host DNA [76]. No samples analysed by MC qPCR exhibited signs of inhibition, suggesting that PCR inhibitors could be efficiently eliminated by the specific isolation and concentration of *T. gondii* DNA. Thus, MC qPCR could be the preferred method when investigating samples prone to PCR inhibition. 

Selection of sample type can also largely affect the probability of *T. gondii* detection. In this study, the heart, as a known predilection site in pigs, small ruminants, horses and poultry [31], and foreleg muscle tissue, as a representative for skeletal muscle, were investigated. Agreement between the DE qPCR analysis of foreleg and heart muscle tissue was moderate to substantial depending on animal species. One wild boar and one roe deer were positive in heart muscle tissue only. Although the representativeness of this comparison is low due to limited sample numbers, this finding may implicate that the occurrence or concentration of *T. gondii* in heart is higher than in skeletal muscle. Additionally, foreleg muscle proved hard to process and yielded only small amounts of pure muscle tissue. Therefore, heart tissue could be the preferred sample type for molecular analysis in epidemiological studies.

In addition to method sensitivity, PCR inhibition and sample type, other aspects should be taken into consideration for the selection of a suitable method for *T. gondii* surveillance in game. Although MC qPCR was the most sensitive, it is also the most cost intensive method. While PD and MC qPCR are both time-consuming, one drawback of the PD qPCR is that samples have to be freshly analysed and consequently must be processed in a short period of time after sampling. Although DE qPCR only allows for the investigation of smaller sample sizes and thus may lack sensitivity, it is the most cost- and time-efficient procedure and allows the screening of frozen tissue samples.

## 5. Conclusions

The results of the present study show that *T. gondii* is present in the sylvatic environment in Brandenburg, Germany, particularly in wild boar. It can be assumed that at least some of the samples that tested positive by direct molecular detection harbour viable parasites and thus could be a source of human *T. gondii* infection. Furthermore, the proportions of positive direct detection results were higher in seropositive wild boar and roe deer than in seronegative animals, indicating a good correlation between seropositivity and the presence of tissue cysts in these game species. However, as some seronegative animals also yielded positive results in direct detection, animals identified as seronegative may still pose a risk for infection when consumed raw or undercooked. To minimize the risk of infection, game should therefore be thoroughly heated (72 °C core temperature, 2 min) before consumption.

## Figures and Tables

**Table 1:** Direct detection of *Toxoplasma gondii* in wild boar and deer.

Game Species	Sample Matrix	Method	Proportion of Positively Tested Animals (%) (No. Positive/No. Tested; 95% CI)	Range of Cq-Values
Wild boar	Foreleg muscle	DE qPCR	7.69 (1/13; 0.19–36.03)	32.09
	Heart	DE qPCR	7.59 (18/237; 4.46–11.74)	30.71–39.68
		PD qPCR	11.72 (15/128; 6.71–18.59)	28.72–37.15
		MC qPCR	13.16 (5/38; 4.41–28.09)	32.21–35.65
		Bioassay	30 (3/10; 6.67–65.25)	na
		Any direct detection method ^a^	11.81 (28/237; 8–16.62)	28.72–39.68
Roe deer	Foreleg muscle	DE qPCR	0 (0/5; 0–52.18)	na
	Heart	DE qPCR	4.79 (7/146; 1.95–9.63)	30.81–39.68
		PD qPCR	3.8 (3/79; 0.79–10.7)	30.07–37.18
		MC qPCR	25 (2/8; 3.19–65.09)	30.81–34.21
		Bioassay	7.69 (1/13; 0.19–36.03)	na
		Any direct detection method ^a^	5.48 (8/146; 2.4–10.51)	30.07–39.68
Fallow deer	Foreleg muscle	DE qPCR	nd	na
	Heart	DE qPCR	0 (0/51; 0–6.98)	na
		PD qPCR	0 (0/42; 0–8.41)	na
		MC qPCR	3.45 (1/29; 0.09–17.76)	31.26
		Bioassay	nd	na
		Any direct detection method ^a^	1.96 (1/51; 0.05–10.45)	31.26
Red deer	Foreleg muscle	DE qPCR	0 (0/7; 0–40.96)	na
	Heart	DE qPCR	0 (0/52; 0–6.85)	na
		PD qPCR	0 (0/31; 0–11.22)	na
		MC qPCR	8.33 (1/12; 0.21–38.48)	38.5
		Bioassay	nd	na
		Any direct detection method ^a^	1.92 (1/52; 0.05–10.26)	38.5

Notes: DE qPCR, qPCR on DNA extracts from 5 g of muscle tissue; PD qPCR, qPCR on DNA extracts from pepsin digest of 50 g heart muscle tissue; MC qPCR, qPCR on DNA from magnetic capture of 50 g of heart muscle tissue; nd, no data; na, not applicable. ^a^ Animals that yielded positive results in at least one direct detection method (positive if at least one of three direct detection methods was applied and tested positive; negative if all direct detection methods applied tested negative).

**Table 2:** Proportion of positive results in direct detection of *Toxoplasma gondii* in seropositive and seronegative wild boar and deer.

Game Species	Method	Proportion of Positive Results among Seropositive Animals in % (No. Positive/No. Seropositive; 95% CI)	Proportion of Positive Results among Seronegative Animals in % (No. Positive/No. Seronegative; 95% CI)	*p*-Value, Fischer’s Exact Test
Wild boar	DE qPCR	36.96 (17/46; 23.21–52.45)	0.52 (1/191; 0.01–2.88)	<0.001
	PD qPCR	61.9 (13/21; 38.44–81.89)	1.87 (2/107; 0.23–6.59)	<0.001
	MC qPCR	50 (3/6; 11.81–88.19)	6.25 (2/32; 0.77–20.81)	0.02
	Any direct detection method ^a^	52.17 (24/46; 36.95–67.11)	2.09 (4/191; 0.57–5.28)	<0.001
Roe deer	DE qPCR	41.18 (7/17; 18.44–67.08)	0 (0/129; 0–2.82)	<0.001
	PD qPCR	37.5 (3/8; 8.52–75.51)	0 (0/71; 0–5.06)	<0.001
	MC qPCR	50 (1/2; 1.26–98.74)	16.67 (1/6; 0.42–64.12)	0.46
	Any direct detection method ^a^	41.18 (7/17; 18.44–67.08)	0.78 (1/129; 0.02–4.24)	<0.001
Fallow deer	DE qPCR	nd	0 (0/51; 0–6.98)	na
	PD qPCR	nd	0 (0/42; 0–8.41)	na
	MC qPCR	nd	3.45 (1/29; 0.09–17.76)	na
	Any direct detection method ^a^	nd	1.96 (1/51; 0.05–10.45)	na
Red deer	DE qPCR	0 (0/4; 0–60.24)	0 (0/48; 0–7.4)	na
	PD qPCR	0 (0/3; 0–70.76)	0 (0/28; 0–12.34)	na
	MC qPCR	100 (1/1; 2.5–100)	0 (0/11; 0–28.49)	0.08
	Any direct detection method ^a^	25 (1/4; 0.63–80.59)	0 (0/48; 0–7.4)	0.08

Notes: DE qPCR, qPCR on DNA extracts from 5 g of heart muscle tissue; PD qPCR, qPCR on DNA extracts from pepsin digest of 50 g heart muscle tissue; MC qPCR, qPCR on DNA from magnetic capture of 50 g of heart muscle tissue; nd, no data; na, not applicable; ^a^ Animals that yielded positive results in at least one direct detection method (positive if at least one of three direct detection methods was applied and tested positive; negative if all direct detection methods applied tested negative).

**Table 3:** Concordance between DE qPCR, PD qPCR, MC qPCR and ELISA measured in kappa values (κ (95% CI)).

Game Species	DE qPCR vs. ELISA ^a^	MC qPCR vs. ELISA ^b^	PD qPCR vs. ELISA ^c^	Direct Detection ^d^ vs. ELISA ^a^
Wild boar	0.47 (0.32–0.63)	0.47 (0.07–0.87)	0.68 (0.49–0.86)	0.59 (0.45–0.73)
Roe deer	0.55 (0.31–0.79)	0.33 (-0.41–1)	0.52 (0.16–0.88)	0.53 (0.28–0.77)
Fallow deer	na	na	na	na
Red deer	na	1	na	0.38 (-0.15–0.92)
Total	0.48 (0.36–0.61)	0.5 (0.21–0.8)	0.61 (0.45–0.77)	0.57 (0.45–0.68)

Notes: DE qPCR, qPCR on DNA extracts from 5 g of heart muscle tissue; PD qPCR, qPCR on DNA extracts from pepsin digest of 50 g heart muscle tissue; MC qPCR, qPCR on DNA from magnetic capture of 50 g of heart muscle tissue; na, not applicable; ^a^ *n* = 486; ^b^ *n* = 87; ^c^ *n* = 280; ^d^ Animals analysed in at least one direct detection method (positive if at least one of three direct detection methods was applied and tested positive; negative if all direct detection methods applied tested negative).

**Table 4:** Concordance between DE qPCR, PD qPCR and MC qPCR measured in kappa values (κ (95% CI)) among individuals analysed by all three methods.

Game Species	DE qPCR vs. PD qPCR	DE qPCR vs. MC qPCR	PD qPCR vs. MC qPCR
Wild boar	0.46 (0.04–0.88)	0.54 (0.09–0.98)	0.68 (0.35–1)
Roe deer	1	0.6 (−0.07–1)	0.6 (−0.07–1)
Fallow deer	na	na	na
Red deer	na	na	na
Total	0.58 (0.22–0.95)	0.47 (0.13–0.82)	0.59 (0.29–0.89)

Notes: DE qPCR, qPCR on DNA extracts from 5 g of heart muscle tissue; PD qPCR, qPCR on DNA extracts from pepsin digest of 50 g heart muscle tissue; MC qPCR, qPCR on DNA from magnetic capture of 50 g of heart muscle tissue; na, not applicable; *n* = 87.

**Table 5:** Statistical analysis and total summary of qPCR results of all animals analysed by all three direct detection methods (*n* = 87).

	Method	qPCR-Negative	qPCR-Positive	*p*-Value, Fischer’s Exact Test
All game species	DE qPCR	84	3	0.048
	50 g qPCR ^a^	76	11	
	DE qPCR	84	3	0.329
	PD qPCR	80	7	
	DE qPCR	84	3	0.132
	MC qPCR	78	9	
	PD qPCR	80	7	0.794
	MC qPCR	78	9	

Notes: DE qPCR, qPCR on DNA extracts from 5 g of heart muscle tissue; PD qPCR, qPCR on DNA extracts from pepsin digest of 50 g heart muscle tissue; MC qPCR, qPCR on DNA from magnetic capture of 50 g of heart muscle tissue. ^a^ Results of PD and MC qPCR combined.

**Table 6:** Proportion of positive results in direct detection of *Toxoplasma gondii* in individuals analysed by using all three direct detection methods in total and in correlation to the serostatus.

Game Species	Method	Proportion of Positive Results in % (No. Positive/ No. Tested; 95% CI)	Range of Cq-Values	Proportion of Positive Results among Seropositive Animals in % (No. Positive/ No. Seropositive; 95% CI)	Proportion of Positive Results among Seronegative Animals in % (No. Positive/ No. Seronegative; 95% CI)
Wild boar	DE qPCR	5.26 (2/38; 0.64–17.75)	35.43–39.68	33.33 (2/6; 4.33–77.72)	0 (0/32; 0–10.89)
	PD qPCR	15.79 (6/38; 6.02–31.25)	29.77–37.15	66.67 (4/6; 22.28–95.67)	6.25 (2/32; 0.77–20.81)
	MC qPCR	13.51 (5/38; 4.41–28.09)	32.21–35.65	50 (3/6; 11.81–88.19)	6.25 (2/32; 0.77–20.81)
	Any direct detection method ^a^	18.42 (7/38; 7.74–34.33)	29.77–39.68	66.67 (4/6; 22.28–95.67)	9.38 (3/32; 1.98–25.02)
Roe deer	DE qPCR	12.5 (1/8; 0.32–52.65)	30.81–39.02	50 (1/2; 1.26–98.74)	0 (0/6; 0–45.93)
	PD qPCR	12.5 (1/8; 0.32–52.65)	30.63–37.18	50 (1/2; 1.26–98.74)	0 (0/6; 0–45.93)
	MC qPCR	25 (2/8; 3.19–65.09)	30.81–34.21	50 (1/2; 1.26–98.74)	16.67 (1/6; 0.42–64.12)
	Any direct detection method ^a^	25 (2/8; 3.19–65.09)	30.63–39.02	50 (1/2; 1.26–98.74)	16.67 (1/6; 0.42–64.12)
Fallow deer	DE qPCR	0 (0/29; 0–11.94)	nd	nd	0 (0/29; 0–11.94)
	PD qPCR	0 (0/29; 0–11.94)	nd	nd	0 (0/29; 0–11.94)
	MC qPCR	3.45 (1/29; 0.09–17.76)	31.26	nd	3.45 (1/29; 0.09–17.76)
	Any direct detection method ^a^	3.45 (1/29; 0.09–17.76)	31.26	nd	3.45 (1/29; 0.09–17.76)
Red deer	DE qPCR	0 (0/12; 0–26.46)	na	0 (0/1; 0–97.5)	0 (0/11; 0–28.49)
	PD qPCR	0 (0/12; 0–26.46)	na	0 (0/1; 0–97.5)	0 (0/11; 0–28.49)
	MC qPCR	8.33 (1/12; 0.21–38.48)	38.49	100 (1/1; 2.5–100)	0 (0/11; 0–28.49)
	Any direct detection method ^a^	8.33 (1/12; 0.21–38.48)	38.49	100 (1/1; 2.5–100)	0 (0/11; 0–28.49)

Notes: DE qPCR, qPCR on DNA extracts from 5 g of heart muscle tissue; PD qPCR, qPCR on DNA extracts from pepsin digest of 50 g heart muscle tissue; MC qPCR, qPCR on DNA from magnetic capture of 50 g of heart muscle tissue; nd, no data; na, not applicable; ^a^ Animals that yielded positive results in at least one direct detection method (positive if at least one of three direct detection methods was applied and tested positive; negative if all direct detection methods applied were negative).

**Table 7:** Genotyping of *Toxoplasma gondii*–positive samples by PCR-restriction fragment length polymorphism (PCR-RFLP) in twelve genetic markers.

Game Species	ID	SAG1	5′-SAG2	3′-SAG2	SAG3	BTUB	GRA6	c22-8	c29-2	L358	PK1	alt. SAG2	Apico	Type
Wild boar	19–171 isolate	II + III	I + II	II	II	II	II	II	II	II	II	II	I	Clonal type II (ToxoDB#3)
	20–528 isolate	II + III	I + II	II	II	II	II	II	II	II	II	II	I	Clonal type II (ToxoDB#3)
	20–531 isolate	II + III	I + II	II	II	II	II	II	II	II	II	II	I	Clonal type II (ToxoDB#3)
	19–201	II + III	I + II	II	II	II	II	II	II	II	II	II	I	Clonal type II (ToxoDB#3)
	18–24	II + III	I + II	II	II	II	II	II	n.d.	II	II	II	II	Incomplete
	19–491	II + III	I + II	II	II	II	II	II	n.d.	II	n.d.	II	I	Incomplete
	19–389	II + III	I + II	II	II	n.d.	n.d. ^a^	II	n.d.	II	II	II	I	Incomplete
	18–92	II + III	I + II	II	II	II	n.d. ^a^	II	n.d.	II	II	n.d.	n.d.	Incomplete
	20–531	II + III	I + II	II	n.d.	II	II	II	n.d.	II	II	n.d.	I	Incomplete
	19–151	II + III	n.d.	II	II	n.d.	n.d. ^a^	II	II	II	II	n.d.	I	Incomplete
	18–85	n.d.	I + II	II	II	n.d.	n.d. ^a^	n.d.	II	II	n.d.	II	I	Incomplete
	17–63	n.d.	I + II	II	II	II	n.d. ^a^	n.d.	n.d.	II	n.d.	II	I	Incomplete
	19–353	II + III	n.d.	II	n.d.	n.d.	n.d. ^a^	II	n.d.	II	II	II	n.d.	Incomplete
	18–133	n.d.	I + II	II	II	II	n.d. ^a^	n.d.	n.d.	II	n.d.	n.d.	n.d.	Incomplete
	19–219	n.d.	I + II	II	n.d.	n.d.	n.d. ^a^	II	n.d.	n.d.	n.d.	II	I	Incomplete
	19–164	II + III	I + II	II	n.d.	n.d.	n.d. ^a^	n.d.	n.d.	II	n.d.	n.d.	I	Incomplete
	19–153	n.d.	I + II	II	n.d.	n.d.	n.d. ^a^	n.d.	n.d.	II	n.d.	n.d.	I	Incomplete
	18–140	II + III	I + II	II	n.d.	n.d.	n.d. ^a^	n.d.	n.d.	II	n.d.	n.d.	n.d.	Incomplete
	19–203	n.d.	n.d.	II	II	n.d.	n.d. ^a^	II	n.d.	II	n.d.	n.d.	n.d.	Incomplete
	20–528	n.d.	I + II	II	n.d.	n.d.	n.d. ^a^	n.d.	n.d.	II	n.d.	n.d.	I	Incomplete
	18–108	n.d.	n.d.	II	n.d.	n.d.	n.d. ^a^	n.d.	n.d.	II	n.d.	n.d.	I	Incomplete
	17–72	II + III	n.d.	n.d.	n.d.	n.d.	n.d. ^a^	n.d.	n.d.	n.d.	II	n.d.	n.d.	Incomplete
	19–481	n.d.	n.d.	II	n.d.	n.d.	n.d. ^a^	n.d.	n.d.	n.d.	n.d.	n.d.	I	Incomplete
	19–171	n.d.	n.d.	n.d.	n.d.	n.d.	n.d. ^a^	n.d.	n.d.	II	n.d.	n.d.	n.d.	Incomplete
	19–378	n.d.	n.d.	n.d.	n.d.	n.d.	n.d. ^a^	n.d.	n.d.	II	n.d.	n.d.	n.d.	Incomplete
	19–161	n.d.	n.d.	n.d.	n.d.	n.d.	n.d.	n.d.	n.d.	n.d.	n.d.	n.d.	I	Incomplete
	19–152	n.d.	n.d.	n.d.	n.d.	n.d.	n.d. ^a^	n.d.	n.d.	n.d.	n.d.	n.d.	n.d.	Incomplete
	17–98	II + III	I + II	II	n.d.	n.d.	III	n.d.	III	II	n.d.	n.d.	I	Incomplete
	17–146	n.d.	n.d.	II	II	n.d.	n.d. ^a^	n.d.	n.d.	II	III	II	n.d.	Incomplete
	17–78	n.d.	n.d.	II	n.d.	n.d.	n.d. ^a^	n.d.	n.d.	II	I	n.d.	I	Incomplete
Roe deer	19–186 isolate	II + III	I + II	II	II	II	II	II	II	II	II	II	I	Clonal type II (ToxoDB #3)
	19–423	II + III	I + II	II	II	II	II	II	II	II	II	II	n.d.	Incomplete
	19–186	II + III	I + II	II	II	n.d.	n.d. ^a^	n.d.	n.d.	II	II	II	I	Incomplete
	17–307	II + III	n.d.	n.d.	II	n.d.	n.d.	n.d.	n.d.	II	n.d.	n.d.	I	Incomplete
	18–147	n.d.	n.d.	n.d.	II	n.d.	n.d.	n.d.	n.d.	n.d.	n.d.	n.d.	I	Incomplete
	18–77	n.d.	n.d.	n.d.	n.d.	n.d.	n.d.	n.d.	n.d.	n.d.	n.d.	n.d.	n.d.	Incomplete
	17–268	II + III	I + II	II	I	n.d.	II	n.d.	II	II	II	II	n.d.	Incomplete
	17–269	n.d.	I + II	n.d.	III	III	III	n.d.	n.d.	n.d.	II	n.d.	n.d.	Incomplete

Notes: n.d., not determined, as no PCR product could be amplified; ^a^ In the nested *GRA6*-PCR, some samples (22/34) showed a PCR-RFLP profile (270 bp and 59 bp) similar to type I, but sequencing the amplicon (329 bp) revealed that this PCR product was not related to *Toxoplasma gondii*.

## Supplementary Materials

The following are available online at https://www.mdpi.com/article/10.3390/microorganisms9081663/s1. Table S1: PCR cycling conditions for PCR-restriction fragment length polymorphism (PCR-RFLP) based *Toxoplasma gondii* genotyping. Table S2: Seroprevalence of *Toxoplasma gondii* in wild boar and deer in total as well as by hunting season, age and sex. Table S3: Results of qPCR of DNA from 5 g of heart and foreleg muscle tissue from the same individuals. Table S4: Detection of *Toxoplasma gondii* in wild boar and deer with at least one direct detection method. Table S5: Cq-values and corresponding dilution of positively tested heart samples of individuals investigated by three direct detection methods. Table S6: Overview of result combinations observed among individuals that tested positive in at least one direct detection method. Table S7: Concordance between DE qPCR, PD qPCR, MC qPCR and ELISA measured in kappa-values (κ (95% CI)) among individuals analysed by all three methods.
