# Supplementary material for: Comparison of Direct and Indirect Toxoplasma gondii Detection and Genotyping in Game: Relationship and Challenges
Source: Microorganisms. 2021 Aug 4;9(8):1663. doi: 10.3390/microorganisms9081663 (PMC8399173; doi:10.3390/microorganisms9081663)
Supplement: Supplementary file 1 [file microorganisms-09-01663-s001.zip › Table S1.pdf]

**Table S1.** PCR cycling conditions for PCR-restriction fragment length polymorphism (PCR-RFLP) based *Toxoplasma gondii* genotyping.

| | **Multiplex Multilocus Nested PCR-RFLP Protocol** | | **Singleplex Nested PCR-RFLP Protocol** | | | |
|---|---|---|---|---|---|---|
| | 1st step: multiplex PCR | | 1st step: singleplex PCR | | | |
| Initial Denaturation | 95 °C, 4 min | | 95 °C, 4 min | | | |
| Denaturation | 94 °C, 30 s | | 94 °C, 30 s | | | |
| Annealing | 55 °C, 1 min | | 55 °C, 1 min | | | |
| Elongation | 72° C, 2 min | | 72 °C, 2 min | | | |
| No. of cylces | 30 | | 35 | | | |
| Final elongation | 72 °C, 5 min | | 72 °C, 5 min | | | |
| | 2nd step: nested PCR | | 2nd step: nested PCR | | | |
| Template | 1.5 µl of 1:2 diluted PCR product of multiplex PCR | | 1.5 µl of undiluted PCR product of singleplex PCR | | | |
| Marker | Apico | all other genetic markers[a] | SAG3 | BTUB, GRA6 | Apico | all other genetic markers[b] |
| Initial Denaturation | 95 °C, 4 min | 95 °C, 4 min | 95 °C, 5 min | 95 °C, 5 min | 95 °C, 4 min | 95 °C, 4 min |
| Denaturation | 94 °C, 30 s | 94 °C, 30 s | 94 °C, 60 s | 94 °C, 60 s | 94 °C, 30 s | 94 °C, 30 s |
| Annealing | 55 °C, 1 min | 60 °C, 1 min | 60 °C, 1 min | 55 °C, 1 min | 55 °C, 1 min | 60 °C, 1 min |
| Elongation | 72 °C, 2 min | 72 °C, 1.5 min | 72 °C, 1 min | 72 °C, 1 min | 72 °C, 2 min | 72 °C, 1.5 min |
| No. of cylces | 35 | 35 | 39 | 39 | 39 | 39 |
| Final elongation | na | na | 72 °C, 5 min | 72 °C, 5 min | 72 °C, 5 min | 72 °C, 5 min |

Notes: DreamTaq DNA polymerase (Thermo Fisher Scientific, USA) was used in singleplex and nested PCRs, while FastStart DNA Polymerase (Roche, Switzerland) was used in multiplex PCR. [a]SAG1, 5′-SAG2, 3′-SAG2, SAG3, BTUB, GRA6, c22-8, c29-2, L358, PK1, alt.SAG2; [b]SAG1, 5′-SAG2, 3′-SAG2, c22-8, c29-2, L358, PK1, alt.SAG2; na, not applied. .
