# Supplementary material for: Comparison of Direct and Indirect Toxoplasma gondii Detection and Genotyping in Game: Relationship and Challenges
Source: Microorganisms. 2021 Aug 4;9(8):1663. doi: 10.3390/microorganisms9081663 (PMC8399173; doi:10.3390/microorganisms9081663)
Supplement: Supplementary file 1 [file microorganisms-09-01663-s001.zip › Table S2.pdf]

**Table S2.** Seroprevalence of *Toxoplasma gondii* in wild boar and deer in total as well as by hunting season, age, and sex.

| Game Species. | Variable | Category | Seroprevalence in % (No. Positive/ No. Tested; 95% CI) | *p*-value, Fischer's Exact Test | OR (95% CI) |
|---|---|---|---|---|---|
| Wild boar | Total | | 20.26 (62/306; 15.9–25.21) | | |
| | Hunting season | 2017/2018 | 24.14 (28/116; 16.68–32.96) | 0.12 | Reference |
| | | 2018/2019 | 25.00 (16/64; 15.02–37.40) | | 1.05 (0.52–2.13) |
| | | 2019/2020 | 14.66 (17/116; 8.78–22.42) | | 0.54 (0.28–1.05) |
| | | 2020/2021 [b] | 10 (1/10; 0.25–44.5) | | na |
| | Age | <1 year | 13.18 (17/129; 7.87–20.26) | 0.004 | Reference |
| | | 1–2 years | 21.14 (26/123; 14.3–29.42) | | 1.77 (0.91–3.45) |
| | | >2 years | 35.19 (19/54; 22.68–49.38) | | 3.58 (1.68–7.62) |
| | Sex | Male | 24.82 (34/137; 17.84–32.92) | 0.09 | Reference |
| | | Female | 16.57 (28/169; 11.3–23.05) | | 0.6 (0.34–1.05) |
| Roe deer | Total | | 10.87 (20/184; 6.77–16.29) | | |
| | Hunting season | 2017/2018 | 15.69 (8/51; 7.02– 28.56) | 0.52 | Reference |
| | | 2018/2019 | 10.81 (8/74; 4.78–20.20) | | 0.65 (0.23–1.87) |
| | | 2019/2020 | 8.16 (4/49; 2.27–19.6) | | 0.48 (0.13–1.7) |
| | | 2020/2021 [b] | 0 (0/10; 0–30.85) | | na |
| | Age | <1 year | 1.52 (1/66; 0.04–8.16) | < 0.001 | Reference |
| | | 1–2 years | 6.98 (3/43; 1.46–19.06) | | 4.88 (0.49–48.49) |
| | | >2 years | 21.33 (16/75; 12.71–32.32) | | 17.63 (2.27–137.04) |
| | Sex | Male | 8.33 (5/60; 2.76–18.39) | 0.61 | Reference |
| | | Female | 12.1 (15/124; 6.93–19.17) | | 1.51 (0.52–4.38) |
| Fallow deer | Total | | 0 (0/80; 0–4.51) | | |
| | Hunting season | 2017/2018 | nd [c] | na [c] | na [c] |
| | | 2018/2019 | nd [c] | | na [c] |
| | | 2019/2020 | 0 (0/58; 0–6.16) | | na [c] |
| | | 2020/2021 [b] | 0 (0/22; 0–15.44) | | na [c] |
| | Age [a] | <1 year | 0 (0/22; 0–15.44) | na | na |
| | | 1–2 years | 0 (0/24; 0–14.25) | | na |
| | | >2 years | 0 (0/33; 0–10.58) | | na |
| | Sex [a] | Male | 0 (0/30; 0–11.57) | na | na |
| | | Female | 0 (0/49; 0–10.25) | | na |
| Red deer | Total | | 6.15 (4/65; 1.7–15.01) | | |
| | Hunting season | 2017/2018 | 7.41 (2/27; 0.91–24.29) | 1 | Reference |
| | | 2018/2019 | 5.00 (1/20; 0.13–24.87) | | 0.66 (0.06–7.81) |
| | | 2019/2020 | 7.68 (1/13; 0.19–36.03 | | 1.04 (0.09–12.66) |
| | | 2020/2021 [b] | 0 (0/5; 0–52.18) | | na |
| | Age | <1 year | 0 (0/28; 0–12.34) | 0.14 | Reference |
| | | 1–2 years | 7.69 (1/13; 0.01–35.42) | | 1.08 (0.93–1.27) |
| | | >2 years | 12.5 (3/24; 2.66–32.36) | | 1.14 (0.98–1.33 |
| | Sex | Male | 6.67 (2/30; 0.82–22.07) | 1 | Reference |
| | | Female | 5.71 (2/35; 0.7–19.16) | | 0.85 (0.11–6.42) |

Notes: nd, no data; na, not applicable; [a] Sex and age of one fallow deer was not recorded and could therefore not be included in the analysis. [b] Due to the COVID-19 pandemic only few samples could be obtained in the 2020/2021 hunting season. Therefore, the 2020/2021 hunting season was excluded from analysis. [c] Fallow deer was sampled only in two hunting seasons.
