# Supplementary material for: Comparison of Direct and Indirect Toxoplasma gondii Detection and Genotyping in Game: Relationship and Challenges
Source: Microorganisms. 2021 Aug 4;9(8):1663. doi: 10.3390/microorganisms9081663 (PMC8399173; doi:10.3390/microorganisms9081663)
Supplement: Supplementary file 1 [file microorganisms-09-01663-s001.zip › Table S3.pdf]

Table S3. Results of qPCR of DNA from 5 g of heart and foreleg muscle tissue from the same individuals.

			DE qPCR Heart Muscle Tissue [a]			κ (95%)
			-	+	total	
DE qPCR foreleg [b]	Wild boar	-	9	1	10	0.62 (-0.04–1)
		+	0	1	1	
	Roe deer	-	3	1	4	na
		+	0	0	0	
	Red deer	-	7	0	7	na
		+	0	0	0	
	Total		19	3	22	0.46 (-0.14–1)

Notes: [a] qPCR on DNA extracts from 5 g of heart muscle tissue; [b] qPCR on DNA extracts from 5 g of foreleg muscle tissue; na, not applicable.
