# Supplementary material for: Comparison of Direct and Indirect Toxoplasma gondii Detection and Genotyping in Game: Relationship and Challenges
Source: Microorganisms. 2021 Aug 4;9(8):1663. doi: 10.3390/microorganisms9081663 (PMC8399173; doi:10.3390/microorganisms9081663)
Supplement: Supplementary file 1 [file microorganisms-09-01663-s001.zip › Table S4.pdf]

Table S4. Detection of *Toxoplasma gondii* in wild boar and deer with at least one direct detection method.

Game Species	Variable	Category	Proportion of Positive Results in % (No. Positive/ No. Tested; 95% CI)	*p*-value, Fischer's Exact Test	OR (95% CI)
Wild boar	Total		11.81 (28/237; 8–16.62)		
	Hunting season	2017/2018	6.49 (5/77; 2.14–14.51)	0.08	Reference
		2018/2019	19.05 (12/63; 10.25–30.91)		3.39 (1.12–10.21)
		2019/2020	11.58 (11/95; 5.92–19.77)		1.89 (0.63–5.68)
		2020/2021 [b]	0 (0/2; 0–84.19)		na
	Age	<1 year	7 (7/100; 2.86–13.89)	<0.001	Reference
		1–2 years	8.51 (8/94; 3.75–16.08)		1.24 (0.43–3.55)
		>2 years	30.23 (13/43; 17.18–46.13)		5.76 (2.1–15.76)
	Sex	Male	14.68 (16/109; 8.63–22.74)	0.23	Reference
		Female	9.38 (12/128; 4.94–15.8)		0.61 (0.27–1.33)
Roe deer	Total		5.48 (8/146; 2.4–10.51)		
	Hunting season	2017/2018	8.33 (3/36; 1.75–22.47)	0.58	Reference
		2018/2019	4.11 (3/73; 0.86–11.54)		0.63 (0.1–4)
		2019/2020	5.41 (2/37; 0.66–18.19)		0.47 (0.09–2.46)
		2020/2021 [b]	nd		na
	Age	<1 year	0 (0/48; 0–7.4)	0.02	Reference
		1–2 years	2.94 (1/34; 0.07–15.33)		1.03 (0.97–1.09)
		>2 years	11.11 (7/63; 4.59–21.56)		1.13 (1.03–1.23)
	Sex	Male	2.08 (1/48; 0.05–11.07)	0.27	Reference
		Female	7.14 (7/98; 2.92–14.16)		3.62 (0.43–30.26)
Fallow deer	Total		1.96 (1/51; 0.05–10.45)		
	Hunting season	2017/2018	nd [c]	na [c]	na [c]
		2018/2019	nd [c]		na [c]
		2019/2020	2.17 (1/46; 0.06–11.53)		na [c]
		2020/2021 [b]	0 (0/5; 0–52.18)		na [c]
	Age [a]	<1 year	0 (0/13; 0–24.71)	0.59	Reference
		1–2 years	0 (1/17; 0.15–28.69)		1.06 (0.94–1.2)
		>2 years	0 (0/21; 0–16.11)		na
	Sex [a]	Male	5.56 (1/18; 0.14–27.29)	0.35	Reference
		Female	0 (0/33; 0–10.58)		0.94 (0.84–1.06)
Red deer	Total		1.92 (1/52; 0.05–10.26)		
	Hunting season	2017/2018	0 (0/25; 0–13.72)	0.49	Reference
		2018/2019	5.88 (1/17; 0.15–28.69)		1.06 (0.94–1.2)
		2019/2020	0 (0/7; 0–40.96)		na
		2020/2021 [b]	0 (0/3; 0–70.76)		na
	Age	<1 year	0 (0/22; 0–15.44)	0.58	Reference
		1–2 years	0 (0/10; 0–30.85)		na
		>2 years	5 (1/20; 0.13–24.87)		1.05 (0.95–1.16)
	Sex	Male	4 (1/25; 0.1–20.35)	0.48	Reference
		Female	0 (0/27; 0–12.77)		0.96 (0.89–1.04)

Notes: nd, no data; na, not applicable; [a] Sex and age of one fallow deer was not recorded and could therefore not be included in the analysis. [b] Due to the COVID-19 pandemic only few samples could be obtained in the 2020/2021 hunting season. Therefore, the 2020/2021 hunting season was excluded from analysis. [c] Fallow deer was sampled only in two hunting seasons.
