# Supplementary material for: Comparison of Direct and Indirect Toxoplasma gondii Detection and Genotyping in Game: Relationship and Challenges
Source: Microorganisms. 2021 Aug 4;9(8):1663. doi: 10.3390/microorganisms9081663 (PMC8399173; doi:10.3390/microorganisms9081663)
Supplement: Supplementary file 1 [file microorganisms-09-01663-s001.zip › Table S5.pdf]

Table S5. Cq-values and corresponding dilution of positively tested heart samples of individuals investigated by three direct detection methods.

ID	Game Species	Serostatus	DE qPCR		PD qPCR		MC qPCR	
			Cq-Value	Dilution	Cq-Value	Dilution	Cq-Value	Dilution
18–092	Wild boar	negative	und.	1:10 dil.	29.8	1:5 dil.	und.	undiluted
18–108	Wild boar	negative	und.	1:2 dil.	36.3	1:5 dil.	35.6	undiluted
18–140	Wild boar	positive	und.	1:2 dil.	34.4	1:5 dil.	und.	undiluted
19–278	Wild boar	negative	und.	1:5 dil.	und.	1:5 dil.	34.4	undiluted
19–378	Wild boar	positive	und.	1:5 dil.	33.7	1:5 dil.	32.2	undiluted
19–481	Wild boar	positive	39.7	1:2 dil.	35,0	1:5 dil.	35.6	undiluted
19–491	Wild boar	positive	35.4	1:2 dil.	37.1	1:5 dil.	34	undiluted
19–275	Roe deer	negative	und.	undiluted	und.	1:5 dil.	34.2	undiluted
19–423	Roe deer	positive	34	undiluted	33.6	1:5 dil.	30.8	undiluted
19–375	Fallow deer	negative	und.	1:5 dil.	und.	1:10 dil.	31.3	undiluted
18–083	Red deer	positive	und.	1:10 dil.	und.	1:10 dil.	38.5	undiluted

Notes: DE qPCR, qPCR on DNA extracts from 5 g of heart muscle tissue; PD qPCR, qPCR on DNA extracts from pepsin digest of 50 g heart muscle tissue; MC qPCR, qPCR on DNA from magnetic capture of 50 g of heart muscle tissue; und., undetermined; dil., dilution.
