# Supplementary material for: Comparison of Direct and Indirect Toxoplasma gondii Detection and Genotyping in Game: Relationship and Challenges
Source: Microorganisms. 2021 Aug 4;9(8):1663. doi: 10.3390/microorganisms9081663 (PMC8399173; doi:10.3390/microorganisms9081663)
Supplement: Supplementary file 1 [file microorganisms-09-01663-s001.zip › Table S6.pdf]

Table S6. Overview of result combinations observed among individuals that tested positive in at least one direct detection method.

ELISA Result	DE qPCR Result	PD qPCR Result	MC qPCR Result	Bioassay Result	Total Number of Animals with This Combination
+	+	+	+	+	0
	+	+	+	na	3
	+	+	na	na	3
	+	na	na	na	13
	+	na	na	+	1
	+	-	na	na	1
	-	+	na	na	5
	-	+	-	na	1
	-	+	+	na	1
	-	-	+	na	1
	+	+	na	+	3
-	+	na	na	na	1
	-	+	+	na	1
	-	+	-	na	1
	-	-	+	na	3

Notes: DE qPCR, qPCR on DNA extracts from 5 g of heart muscle tissue; PD qPCR, qPCR on DNA extracts from pepsin digest of 50 g heart muscle tissue; MC qPCR, qPCR on DNA from magnetic capture of 50 g of heart muscle tissue; na, not applied.
