# Supplementary material for: Comparison of Direct and Indirect Toxoplasma gondii Detection and Genotyping in Game: Relationship and Challenges
Source: Microorganisms. 2021 Aug 4;9(8):1663. doi: 10.3390/microorganisms9081663 (PMC8399173; doi:10.3390/microorganisms9081663)
Supplement: Supplementary file 1 [file microorganisms-09-01663-s001.zip › Table S7.pdf]

Table S7. Concordance between DE qPCR, PD qPCR, MC qPCR and ELISA measured in kappa-values (κ (95% CI)) among individuals analysed by all three methods.

Game Species	DE qPCR vs. ELISA	MC qPCR vs. ELISA	PD qPCR vs. ELISA
Wild boar	0.46 (0.04–0.88)	0.47 (0.07–0.87)	0.6 (0.25–0.96)
Roe deer	0.6 (-0.07–1)	0.33 (-0.41–1)	0.6 (-0.07–1)
Fallow deer	na	na	na
Red deer	na	1	na
Total	0.47 (0.13–0.82)	0.5 (0.21–0.8)	0.59 (0.29–0.89)

Notes: DE qPCR, qPCR on DNA extracts from 5 g of heart muscle tissue; PD qPCR, qPCR on DNA extracts from pepsin digest of 50 g heart muscle tissue; MC qPCR, qPCR on DNA from magnetic capture of 50 g of heart muscle tissue; na, not applicable; n = 87.
